# Vulcano: A new robotic challenge for legged robots

**DOI:** 10.3389/frobt.2022.1057832

**Published:** 2023-01-25

**Authors:** Manuel Domingos, Francisco Pedro, Alberto Ramos, Matthias G. Funk, Armando Mendes, José Cascalho

**Affiliations:** ^1^ Escola Básica e Integrada de Rabo de Peixe, Ribeira Grande, Portugal; ^2^ Escola de Novas Tecnologias dos Açores (ENTA), Ponta Delgada, Portugal; ^3^ LIACC FCT University of the Azores, Ponta Delgada, Portugal

**Keywords:** legged robots, whegs, robotic competition, educational robotics, volcanic scenario

## Abstract

The *Vulcano* challenge is a new and innovative robotic challenge for legged robots in a physical and simulated scenario of a volcanic eruption. In this scenario, robots must climb a volcano’s escarpment and collect data from areas with high temperatures and toxic gases. This paper presents the main idea behind this challenge, with a detailed description of the simulated and physical scenario of the volcano ramp, the rules proposed for the competition, and the conception of a robot prototype, *Vulcano*, used in the competition. Finally, it discusses the performance of teams invited to participate in the challenge in the context of Azorean Robotics Open, the Azoresbot 2022. This first test for this challenge provided insights into what the participants found exciting and positive and what they found less positive.

## 1 Introduction

Robotics has been used to promote formative and educational activities regarding the development of STEAM (science, technology, engineering, art, and mathematics). These activities are found at all educational levels, from kindergarten to high school (Benitti, 2012); Kubilinskiene et al., 2017; Viegas and Villalba, 2017; Chaldi and Mantzanidou, 2021. They are seen as fostering students’ motivation and interest in further study of science and technology, as Lauwers et al., 2009 suggest.

At the same time, educational robotic activity has been associated with robotic competitions. An example of these initiatives is the RoboCup and RoboCupJunior, where challenges of different types have been proposed, like in Asada and von Stryk, 2020. Some of these challenges involve developing a robot, either from scratch or starting with an off-the-shelf platform, and forcing teams to follow the competition rules. These activities motivate and give hands-on experience, forcing students to solve unexpected problems, as demonstrated in Ribeiro and Lopes, 2020.

However, Kaloti-Hallak et al., 2015 have concluded in their study that although competitions are effective in achieving meaningful learning in robotics and computer science concepts, competitions also have less desirable effects. On the positive side, the authors refer to technological and personal skill development. On the negative side, they state that “learning opportunities were pushed aside in favour of constructing robots that tried to accomplish the mission” (Kaloti-Hallak et al., 2015), p.111). Nevertheless, worldwide, the number of robotic competitions has been growing. As mentioned in Brancalião et al., 2022, growth implies attracting more students for technological areas. In competitions focused on education, the authors state that they have “objectives focused on encouraging young students to pursue careers in STEM areas, develop skills, teach how to work in a team, assist teachers and universities in multidisciplinary domains and expose students to real problems, solving and practical application of their knowledge” (p.33).

A complementary approach to foster the use of robotics is to use simulation (e.g., competitions in the RoboCup initiative). Nowadays, these activities are widespread, mainly to provide an accelerated, safe, and fully controlled virtual testing and verification environment (Choi et al., 2021). Moreover, considering the benefit of using simulation for testing and providing essential information regarding improvements in robotic behaviors, there are competitions where both simulation and reality co-exist (e.g., Robot@Factory Lite, Braun et al., 2020).

Finally, some competitions have promoted societal challenges, also mentioned as application leagues in Ferrein and Steinbauer (2016). These competitions are intended to be applied to societal and economic relevant problems. There are several examples of these challenges. One of them is the RoboCup initiative related to the rescue initiative (Asada and von Stryk, 2020). However, there are others, such as the Fire-Fighting Robot International Competitions (Ahlgren and Verner, 2001).

In Table 1, we provide an overview of active (or outstanding) competitions in application leagues and their characteristics, with the reference to the *Vulcano* competition in the last row. The table shows the target groups, the themes or scenarios, some skills participants must have or acquire, the robot’s locomotion, and if the robots are autonomous or remote-controlled. The target corresponds to different ages of participants, with *M* for middle school (11–14 years old), *Sc* for secondary (14–18 years old), and *Sn* for senior. Skills (technical) are identified as (robot) building (*Bd*), mechanics (*Mc*), programming (*Pg*), and/or higher level skills (*Hl*) applied for competitions where robots must use localization and mapping, computer vision, or artificial intelligence; scenarios characterize the arena where robots compete, such as fixed (*Fx*), with flat (*Fl*), sloping (*Sp*), and/or uneven (*Un*) grounds; grasping (*Gp*) if the robot must grasp and/or transport objects; and/or real (*Re*) if scenarios are close to reality; locomotion, such as wheels (Wh), legs (*Lg*), whegs (*Wg*), or caterpillar (*Cp*), with a “c:” if it is the standard option and identifying if there is a predefined type (*PD* or *nPD*); and, finally, autonomy, featuring whether the robot is autonomous or remote-controlled, sometimes linked to a specific target.

**TABLE 1:** Competitions for different scenarios in educational robotics.

Competition—challenge	Target	Skills	Scenarios	Locomotion	Autonomy
National Robotics Challenge—Rescue	M; Sc	Bd; Mc; Pg	Fx; Fl; Sp	nPD; c: Wh	No
RobotCupRescue Line and Maze	Sc; Sn	Bd; Mc; Pg	Fx; Fl; Sp	nPD; c: Wh	Yes
Portuguese Rob. Open - Robot@Factory Lite	Sn	Pg; Hl	Fl; Re	Wh	Yes
Port. Rob. Open—Robot@Factory Lite	Sc; Sn	Bd; Pg	Fx; Fl; Gp	nPD; c: Wh	Yes
Eurobot Contest	M; Sc; Sn	Bd; Mc; Pg	Fx; Fl; Gb	nPD; c: Wh	Yes (Sn)
IROS Competition—Minesweepers	Sc; Sn	Bd; Mc; Pg; Hl	Sp; Un	nPD	Yes (Sn)
Firefighting Home Robot Contest	M; Sc; Sn	Bd; Mc; Pg	Fx; Fl	nPD; c: Wh	Yes (Sn)
Azoresbot—Vulcano competition	M; Sc	Bd; Mc; Pg	Fx; Sp; Un	Wh; Wg; Lg; c: Wg	Yes

Target: P for primary education (5–11 years old), M for middle school (11–14 years old), Sc for secondary (14–18 years old), Sn for senior. Skills: Bd, building; Mc, mechanics; Pg, programming, and Hl, higher level. Fx, scenarios: fixed, Fl, flat; Sp, sloping; Un, uneven; Gp, grasp; Re, real. Locomotion: PD, predefined; or nPD, not predefined; c:, most common; Wh, wheels; Lg, legs; Wg, whegs; or Cp, caterpillar.

In the first rows, we display two different contests where the theme of rescue is addressed. The first is the National Robotics Challenge (NRC), a competition in the United States . In this challenge, robots are not autonomous. They have to move in a predefined arena and pick up four colored ping-pong balls from four holding device (pick pylons) locations and place them into a receiving jig (drop pylons). A camera provides visual information to the team when the robot is inside a tunnel. In a predefined scenario, two teams compete at the same time. This competition has similarities with the RoboCup rescue line and maze. In both, victims must be identified to be rescued. The main difference is that in the RoboCup rescue, robots are autonomous. In the rescue line, the robot must follow a line in a previously predefined circuit, while in the rescue maze, it must discover the victims in a maze that must be explored. Both challenges address the building of the robot and programming skills using different types of sensors. Furthermore, these challenges are replicated in different challenges, such as in the Portuguese and Brazilian Robotics Open (see Portuguese Society of Robotics and Brazilian Robotics Olympiad).

Another competition related to social challenges is the *RoboCup@Home*, which addresses the area of service and assistive robot technology with high relevance for future personal domestic applications. There are three leagues in this challenge. The table presents the two that use standardized robots, the Domestic Standard Platform League (DSPL) and the Social Standard Platform League (SSPL). The main difference from the other challenges is that the target skills are mostly related to programming for high-level tasks. The robots have to plan, identify objects, interact with persons, and navigate in natural environments. Competitions of another type are those related to industrial environments.

Robot@Factory, part of the Portuguese Robotics Open competition, aims to use robots in an environment to transport materials between warehouses or machines that process those materials. The Robot@Factory Lite competition is conducted in a predefined arena of 1.7 × 1.2 m. The Robot@Factory 4.0 version has the same goals but with a more challenging environment (e.g., it uses ArUco IDs on the floor to guide the robot, instead of a line). Robot@Factory Lite provides an easy-to-build scenario, a robot prototype, and also a simulator, making it a very interesting tool for teams starting to learn about robotics.

The Eurobot competition specifies a different scenario for each competition year. The robots are created by the teams. They have to find objects while moving around the scenario and have to grasp plastic pieces identified with patterns. The robots are autonomous only for the senior teams. The most interesting feature in this competition is the fact that the robots have to detect and manipulate pieces, increasing the complexity associated with the mechanics and programming of the robots.

Several contests are usually proposed in the context of the IEEE International Conference on Intelligent Robots and Systems (IROS), which also change from year to year. We selected from IROS (2019), the Minesweepers competition. The robots are placed in a fenced indoor arena with a size of 10 m × 10 m, simulating rough terrain with some steep inclines and some trees as obstacles. The setting has both buried and surface mines, simulating a real-life scenario for minesweepers. In the junior championship, the robots are not autonomous. This competition is an example where the scenario is very close to a real scenario, where this type of robots can be experimented.

Finally, in the Trinity College Firefighting Home Robot Contest (FHR), discontinued recently, the goal is to extinguish candles and avoid obstacles. A maze, similar to that in the more recent RoboCup rescue challenges, must be traversed to find the candle. The robots in this competition are also autonomous. FHR and NRC are the oldest robotics competitions, with 28 and 37 editions, respectively.

The main objective of the *Vulcano* challenge is to provide a competition that can teach aspects related to STEAM themes. It is a new challenge for robots with legs (or whegs) that proposes a volcanic eruption as an inspiring scenario. In this environment, the robots must climb the volcano’s slopes and collect data from areas with high temperatures and toxic gases, unbearable for humans. This new competition introduces the following innovative and challenging aspects:

• Provides a scenario where robots are asked to use legs (or whegs) rather than wheels.
• The robot faces uneven and rough terrain, an environment quite different from the challenges where the ground is flat and/or uses adapted ramps.
• There is an increasing difficulty in using sensor measurements due to the oscillations suffered by the robot along its movement.

The main differences between this proposal and the other competitions, depicted in Table 1, are the use of legs and the uneven and rough terrain. There are other competitions with some of those aspects, like the rough terrain, as in the minesweepers. However, as far as we know, this is the only competition that tries to simulate active volcano climbing. At the same time, this competition is related to the history and heritage of islanders living in a volcanic region. This challenge was first proposed as one of the challenges of the Azorean Robotics Open, Azoresbot 2022, the festival of robotic competitions in the Azores.

The paper is organized as follows: In the Section 2, we explain the robots’ configuration, the ramp, and the rules of the competition. In the following section, we present the results of the Azoresbot 2022 challenge experience and from a survey answered by the participants. We also present the development of a simulation to train participants in this challenge. Finally, the article ends with conclusions and future work.

## 2 Material and methods

In this section, we will first detail the rules of the proposed competition. Then, we will present the robot Vulcano prototype created for the challenge. This prototype was tested in the scenario and provided a first model for teams participating in the Azoresbot 2022. Finally, we will discuss the simulated scenarios created in the Webots platform (https://www.cyberbotics.com/) as an initial step for creating a complementary simulated competition.

### 2.1 Rules of the Vulcano competition

In the first version of the Vulcano contest, the robots had to climb a ramp without exceeding a certain limit, detect a color that corresponds to the places where the data should be collected, and then return to the starting point within a maximum time interval. The scenario has an infrared source in the range of 700 nm–1,100 nm at the top of the ramp. This source serves as the orientation point when the robots go up the ramp.

The rules are as follows:

i. The robot has to execute all tasks in a maximum time of 5 min.
ii. It has to climb the ramp from the designated blue starting point.
iii. It should not surpass a given return line to avoid its destruction.
iv. Therefore, it must detect color (s) on the final part of the ramp.
v. It has to return to the starting area.

Successfully executing these four tasks gives the team five points. The best score establishes the ranking. In the case of a draw, the execution time (till the last successful task) will be the decisive factor.

The robot has to use sensors to find obstacles on the ramp, mainly because it has to avoid them, as they are too big to overcome. The smaller obstacles would be resolved by adequate whegs. Therefore, the teams had to calibrate the sensor due to the capacity of the whegs.

Although there was the possibility of using a temperature sensor to prevent the robot from getting too close to the volcano, it was decided to recognize the minimum distance to the infrared light source by using the colors on the ground.

As part of the rules, teams can compete even if they complete only part of the proposed tasks. For example, a team scores for a safe return even if it cannot detect the colors on the ground. This strategy gives the teams the opportunity to test the different parts of the challenge separately and to participate, even if the robot cannot complete all the proposed tasks. The rules for this first edition were purposely simplified taking into account the unfamiliarity of the participating teams’ ability to meet the challenges proposed in the ramp climb.

**FIGURE 1:**
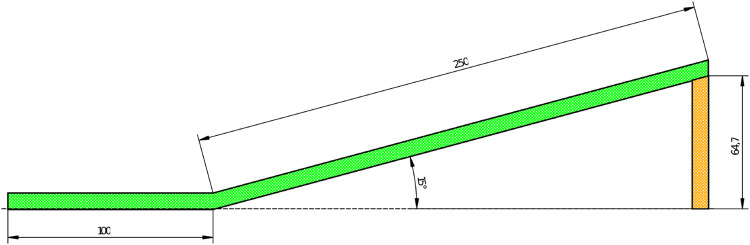
Ramp dimensions (in centimeters).

Figure 1 depicts the proposed ramp dimensions. At the top of the ramp, we can find the light bulb. On the other side is the initial position (where the robots will start the challenge). This competition was designed for teams aged 13+ having four participants.

In this section, we will first detail the rules of the proposed competition. Then, we will present the robot Vulcano prototype created for the challenge. This prototype was tested in the scenario and provided a first model for the teams participating in the Azoresbot 2022. Finally, we will discuss the simulated scenarios created in the Webots platform (https://www.cyberbotics.com/) as an initial step for creating a complementary simulated competition.

### 2.2 Learning goals

Volcanic environments are tough for robots (Borgese et al., 2021). The use of robots on a rough ground where it is difficult to move, avoid obstacles, and try to achieve a target is one of the project’s main goals. Another objective is the explicit use of legs or whegs to achieve this task.

One of the difficulties concerning the task is keeping the robot on target. The movement using the whegs will balance up and down and to left and right. The robot must use an ultrasonic sensor to avoid big obstacles.

At the same time, it must detect the direction where the light is stronger. The other difficulty is finding the way back to the initial area and descending the ramp. Finally, detecting colors on the ground could be tricky because of the interference from the light radiator.

The main immediate learning goals were settled to be the following.

• Learn to program the movement of the robot.
• Tuning the robot’s activity using the sensors.
• Redesign some of the components (e.g., the whegs of the robot).
• Fine-tuning of the new physical components.

### 2.3 Robot development

Vulcano is a four-wheel differential drive robot. The main approach for the development of this robot was based on the DIY (do it yourself) concept with affordable and simple hardware. To achieve this, a very simple frame was designed using CAD software, in this case FreeCad, and printed in an FDM 3D printer using PLA (polylactic acid) filament (see Figure 5). All parts of the frame can be improved by the teams during the competition. Hardware was chosen based on choices made in the development of previous robots created in the Azores, namely, the Azoresbot V2 (Cascalho et al., 2021) and Azbot1C (Pedro et al., 2021). This allowed the teams to approach the challenge with some basic knowledge regarding micro-controller programming and the use of sensors and actuators.

We chose to use the ESP32 micro-controller, in our case the ESP32-DEVKITC-32E board and a DRV8833 two-channel H-bridge motor driver, driving four DC motors, two on each side of the robot connected in parallel. The power supply is based on two 18,650 batteries protected by a battery management system. A simple voltage regulator was built based on a low-dropout positive regulator, NCP1117, in order to provide the 5 V needed to power all electronic components. Table 2 depicts the hardware components of the robot.

**TABLE 2:** Hardware components for the robot Vulcano.

Qty	Description
1	ESP32-DEVKITC-32E
1	DRV8833 H-Bridge
4	DFRobot 6 V micro DC motor with encoder
3	Flame Sensor (IR receiver + LM393)
1	Color Sensor TCS 3200
2	MR18650 batteries
1	WH-2S80A BMS
1	NCP1117 5 V

### 2.4 Ramp development

**FIGURE 2:**
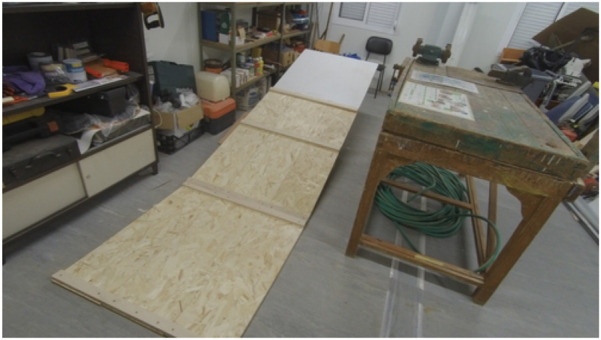
Wooden structure of the ramp.

**FIGURE 3:**
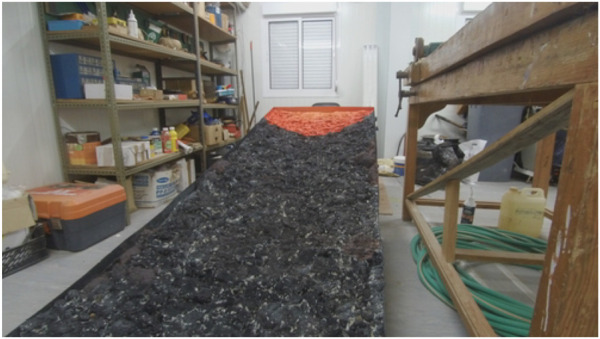
Final ramp for Vulcano challenge.

The structure of the ramp for the challenge was entirely built of wood. It is almost 1 m wide and 3 m to 5 m long with an angle of approximately 15° (Figure 2). The surface was covered with polyurethane foam in several layers molded to give the shape of rocks on a volcanic slope. This surface was painted not only to give some realism to it but also to delimit the action zones of the robot—dark where is no danger and yellow, orange, or red where the danger is present. The final appearance of the ramp is depicted in Figure 3.

On the top side of the ramp, there is a heater radiator used as an infrared source in the range of 700 nm–1,100 nm. We used an electric infrared heater with three quartz lamp elements. Robots use this source as a means of getting a point of orientation when climbing the ramp.

## 3 Results

**FIGURE 4:**
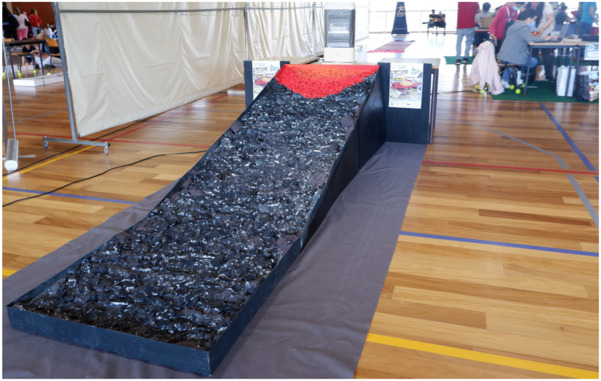
Challenge ramp in the Azoresbot 2022.

**FIGURE 5:**
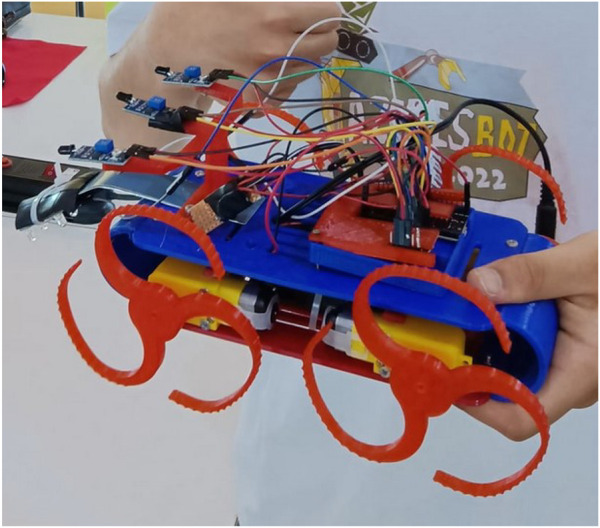
Vulcano robot in the Azoresbot 2022.

The Vulcano challenge was conducted in June 2022 in Ribeira Grande, Azores, during the 2nd Azorean Robotics Open, Azoresbot 2022 (see Figures 4, 5). This festival was an opportunity to test the ramp, the robot, and the rules of the competition. The competition was held for four teams, each with three students and a tutor. We also had two different age groups, ages under 15 and 15+, and although the goals were the same for both groups, they competed separately. A kit box was delivered to each team with the material to assemble the robot.

The rules used in the challenge are described in Table 4. In addition to the division between senior and junior teams, the rules indicate which characteristics the robots participating in the competition must comply with and the maximum number of attempts by each team. Along with the rules available on a web page, each team received a kit manual for building the robot. The *stl* files and code samples were also provided in a public repository https://robotics-and-ai-group-of-uac.github.io/Vulcano/. With the kit, we also wanted to avoid some problems that the teams may face. For example, we put aside the so-called breadboard since it did not present effective and reliable connections between the different components, leading to constant failures, both in the sensors and the controllers themselves, and resorted to other solutions. We gave the teams the possibility to use blocks of electrical quick connectors. It should be noted that we also gave the option of welding to all teams even if they had never performed it before.

**FIGURE 6:**
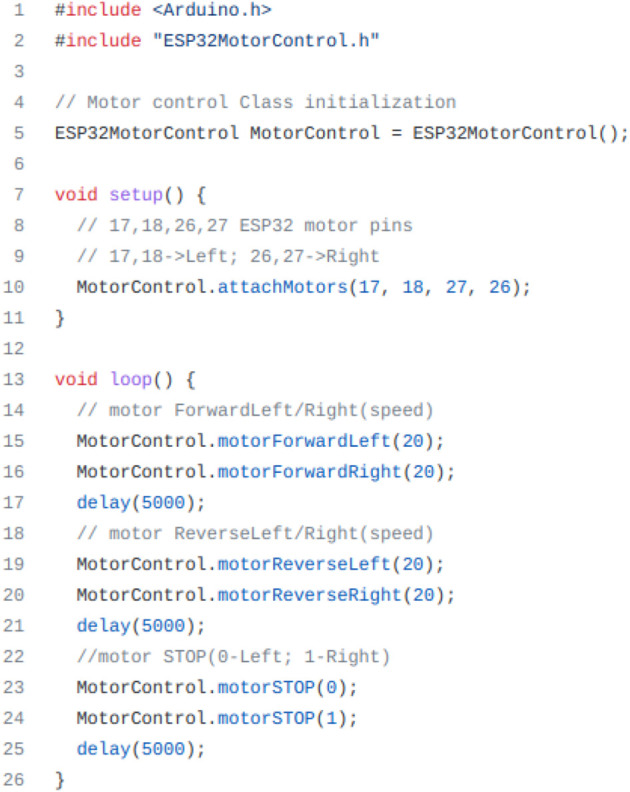
Sample code to test motors.

**FIGURE 7:**
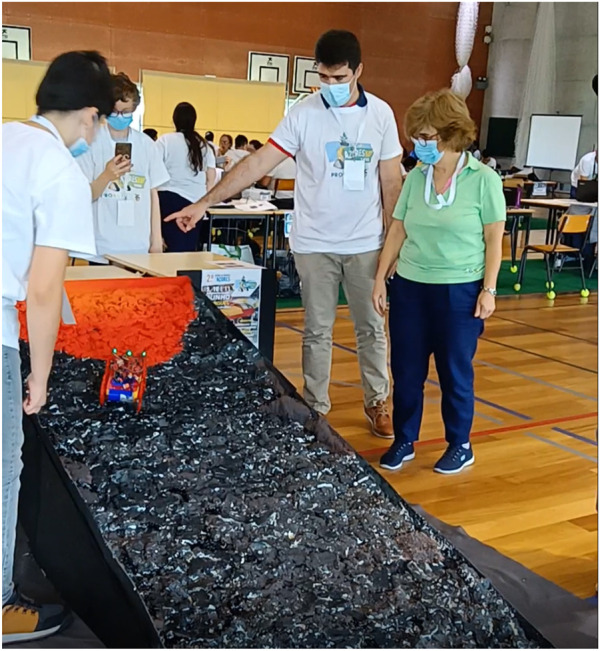
One of the teams testing the robot.

Some generic code samples to test the sensors and the motors (Figure 6) were also made available, but the final programming of the robot was part of the tasks each team had to accomplish. In the end, all teams assembled the robot and programmed and tested it on the ramp, as depicted in Figure 7.

**FIGURE 8:**
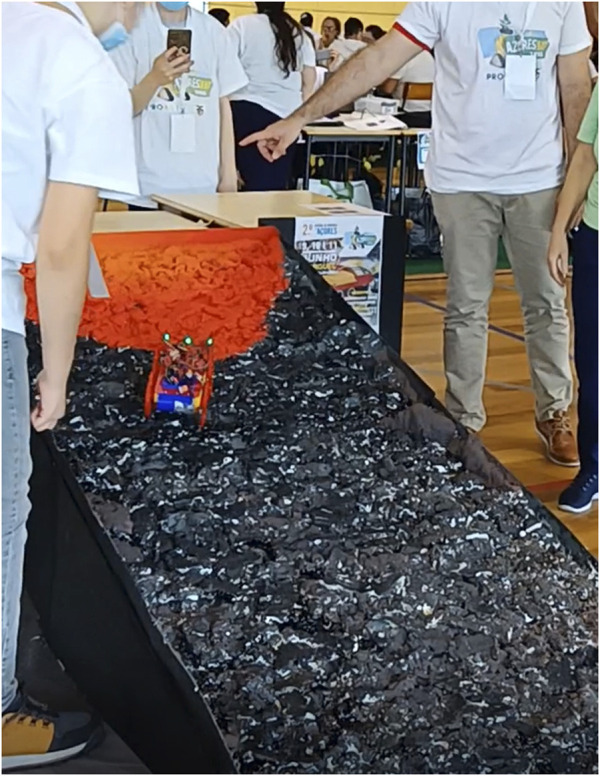
Wheg created by one of the teams.

Given the importance of combining the educational value of 3D printing with the fun side of creation Van et al. (2016), three FDM 3D printers were available during the competition so that teams could produce the parts they wanted to improve. Although only one team took advantage of this resource to produce different types of whegs and test them (see Figure 8), other teams had the opportunity to have the first contact with this technology when it was necessary to replace the broken parts of their robot.

Each team was given 2 days to complete all the described phases, from assembly to programming and testing the robot. The third day was dedicated to the competition, where each team presented its work with the possibility of some final adjustments.

A simple questionnaire was made available to all teams to collect feedback on the challenge. Since only four teams participated, the sample is small but essential at this stage of the challenge development.

The teams, in general, performed well and fulfilled all the proposed activities. Those activities not only included the tasks mentioned in the description of the challenge but also included the assembling of the robot and programming it. They all stated that they enjoyed their participation and would participate in the competition again.

In the questionnaire, when asked to identify positive aspects of the event, we noticed that the most relevant keywords were learning, cooperation, and knowledge, as depicted in Table 3. Therefore, although it was a competition, this was not even mentioned by the students. It is important to refer that this was an open question, and the students could choose any word they wanted.

**TABLE 3:** Most relevant keywords for positive aspects.

Key words	%
Learning	42
Cooperation	25
Knowledge	17
Challenge	8
Easy chassis construction	8

**TABLE 4:** Rules for the Vulcano competition in the Azoresbot 2022.

Rules for Vulcano competition (2022)	
R.1	Teams can be made up of a maximum of five students and a tutor
R.2	The teams can be *senior* or *junior* according to the average age of their members, excluding the tutor. The minimum age of the team members is 9 years: Junior—the average age of the team cannot exceed 15 years; Senior—the average age of the team is over 15 years
R.3	The robot used must have a maximum length of 30 cm and a width of 25 cm and must be completely autonomous
R.4	The organization provides a standard robot that can be modified by the teams during the assembly period and during the competition period
R.5	No commercial robots or robotic kits in which the participants have not participated in their development will be allowed
R.6	The robot can be equipped with any type of wheel, wheg, or a mixture of the two. Systems using caterpillars or any type of commercial wheel or wheg will not be allowed. Robots using legs are allowed, provided these have been produced by the teams themselves
R.7	The robot may be equipped with any type of sensor that allows it to guide its movement on the ramp. The robot must have a color sensor and an LED
R.8	Each team can make a maximum of three attempts to complete the competition, choosing at the end which one it wishes to validate. The score of each attempt does not accumulate with any of the other attempts
R.9	Each attempt will have a maximum time of 5 min

**FIGURE 9:**
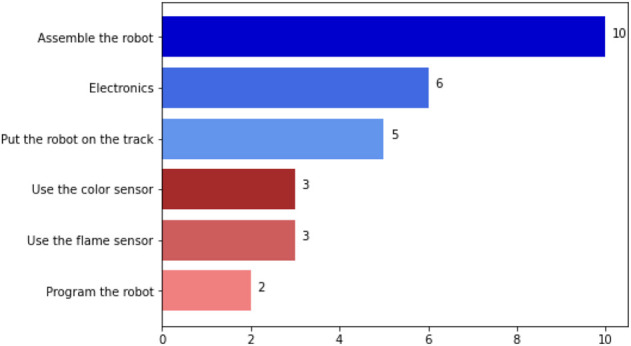
Number of answers to the question about the easiest activities for students in the Vulcano challenge.

From the students’ answers, when asked about the challenge activities, the assembly of the robot chassis was considered the easiest, as depicted in Figure 9. The simplicity of the robot chassis and its assembly allowed other more complex challenges to be accepted, namely, in terms of component connections. Therefore, although the teams had some difficulties, mainly with some electronic components, it was considered an easy activity. The most serious difficulty with the electronic components was related to the reading of the color sensor values. The permanent oscillation movement of the robot on the ramp did not allow a constant distance from the ground, which made the reading of this sensor difficult. Another problem had to do with the reading through infrared sensors. The radiator’s position at the top of the ramp was not optimal, and the teams also had difficulty using these sensors to steer the robot. Almost half of the participants mentioned the difficulty in keeping the robot on the right path (Figure 9).

**FIGURE 10:**
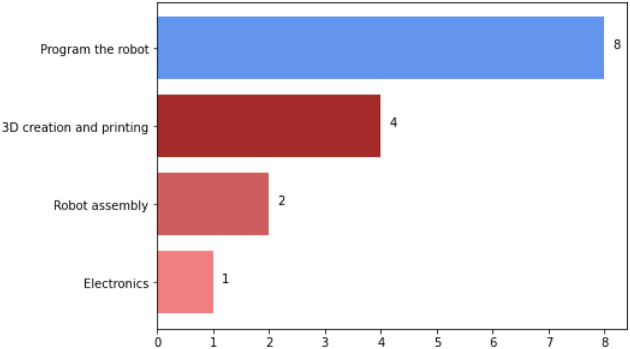
Number of answers to the question about the most difficult tasks in educational robotics for students.

When asked in general what the most difficult tasks in the activity were, the students answered ‘programming the robot’ (Figure 10). We acknowledged that this was the most challenging task for them during the festival, primarily because of a lack of previous knowledge in programming, namely, in C language.

It is important to notice that “Program the robot” corresponds to the initial programming activity. This initial program had to be adjusted several times until the teams could effectively “use the color sensor” and “use the flame sensor”. In the later activity, not only was the program adjusted but even changes in the robot’s structure were tested.

## 4 Discussion

The main goal of the first interaction with the robot prototype and the environment created was to understand if the challenge was suitable to the age of the participants, their main difficulties, and the teams’ success in fulfilling the different tasks of the challenge. All teams completed the challenge comprising assembling, programming, and moving the robot up and down the ramp. These facts demonstrated the success in completing these tasks (see Figure 9). The rules were set in the most simplified way possible from the beginning by the promoters of the challenge because it was assumed that in the first edition, the teams should complete the task. In this challenge, participants were using for the first time the robots with sensors in a complex environment (i.e., uneven terrain and direction detected through infrared radiation). The participants were asked to complete the up and down task in 5 min after the assembly and programming activities. In the end, the teams managed to get the robot up and down the ramp in less than 1 min. One of the teams completed the challenge without reading the red color on the ground, simply by adjusting the speed and counting the time needed to reach the top. Among the participants, at the time of competing, only one team used the fire sensor to direct the robot to the target. The fact that teams were allowed to test the robot’s performance without many restrictions allowed a more vast space for experimentation, which also contributed to the more active participation of the teams. One of the revealing signs of this interest is that one of the teams tried to improve the robot’s performance by designing new whegs, using one of the 3D printers during the competition (see Figure 8). It should be noted that this situation also fostered sharing among the teams, raising the question of the role of a purely competitive event *versus* an event with a more pronounced component of cooperation. The jury accepted the evidence of all the teams as valid. They only monitored the movement of the robot. In fact, the teams were not required to use a way of checking whether the robot detected red or used the fire sensor (e.g., by using LEDs or signaling it with a beep). These results were discussed afterward and led to new ideas about the rules for future editions. The simplest were that juries need to know whether the robots are collecting data, setting milestones in the tasks to be performed, and whether the robots are using this data to make decisions. When it comes to evaluation, it is to be expected that data collection should be taken into account in the final performance of the robots, ensuring good scores for teams that consider the data collected and not just the speed of the robots. These and other suggestions are compiled below, presenting some of the weaknesses and proposals for future editions of the competition.

In a nutshell, the weaknesses identified are as follows.

• The source of infrared radiation: The source should be more protected from ambient light and directed toward the robots. Otherwise, it is difficult for the robots to use sensors and detect the radiation with increasing intensity as they approach it. We intend to build our own radiator using the same type of thermal elements in a more compact and organized way to optimize the use of infrared sensors.
• The color sensors: Because the robot oscillates as it moves, it was difficult for the teams to calibrate the color sensor correctly with the type of sensors used. A new sensor based on a different chip (e.g., VEML6040) will be tested to see if the performance improves.
• Undefined milestones: Regarding the tasks performed by the robots, it would be interesting if the teams pointed out the detection of some special points in the environment (e.g., the red point that simulates a hot spot where the robot should not cross) as milestones of the challenge.
• Lack of information (for jury evaluation): Performance should be measured not only based on the time used to finish the tasks but also on how these tasks were performed. To this end, some additional information should be included about how the robots are performing the task (e.g., sending data or collecting data to be present throughout and after the task execution). This additional information should be included in the jury’s final evaluation of the robots’ performance.
• Challenge too easy: The promoters decided to keep the rules easy to follow to enable the teams to succeed in the tasks. In this case, all teams completed the task in less than 1 min. Therefore, to keep it interesting, the challenge should become more complex. A proposal is to add new elements to the ramp that could force teams to use other types of sensors, such as temperature and humidity, or even make the ramp longer.
• Lack of simulators to help teams test and improve algorithms: Nowadays, simulators are increasingly considered an essential step in robotics, mainly for verification and validation (Choi et al., 2021; Afzal et al., 2022). The intention to create a simulator of the robot and the scenario was discussed from the beginning of the challenge implementation. Initial efforts were made using Webots.

In the following subsections, we detail the last two proposals in the context of future work.

### 4.1 Increasing the complexity of the challenge

Regarding the increase in the complexity of the challenge, we expect to add to the scenario a richer environment where the robots must use more sensors to accomplish all the tasks. The addition of some obstacles implies the use of a distance sensor. Another possible sensor to add to the robot could be the temperature sensor. This sensor could be based on the TMP36 to determine the ambient temperature or based on the MLX90614 to evaluate the temperature at specific points on the ramp. The humidity sensor could also be considered, using a vaporizer, e.g., a water-based vaporizing machine, to simulate fumaroles on the ramp, creating different humidity conditions in different areas.

Finally, we could improve the efficiency of the challenge by implementing communication between the robot and a local server using the Message Queuing Telemetry Transport (MQTT) protocol. The sensor data acquired by the robot can be displayed in this way, creating a more dynamic environment in the challenge, both for the teams and the public. Naturally, it is hoped to have more teams testing the challenge so that findings on its use by different teams can contribute to a more informed appreciation of the challenge.

### 4.2 Robot and ramp simulation

**FIGURE 11:**
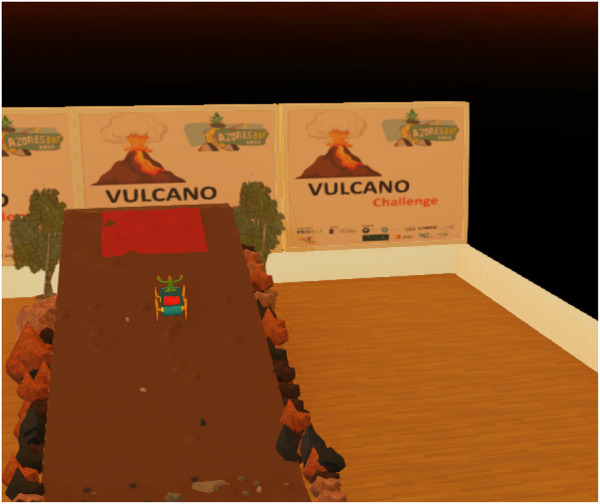
Simulated Vulcano world with the Vulcano robot picture on the ramp. The scenario on the back reproduces the Azorean Robotics Open poster.

Robotic simulations are commonplace today, primarily to provide an accelerated, safe, and fully controlled virtual test and verification environment (Choi et al., 2021; Afzal et al., 2022). They are widely used for most sophisticated and complex robots as a way to obtain large amounts of training data for machine learning on low-cost budgets. However, they are also crucial for testing in robotic competitions. An example of mixing these two worlds is Robot@Factory Lite (Braun et al., 2020). Considering these aspects and the fact that the Vulcano challenge has an added difficulty regarding the construction of the ramp, we decided to create a virtual challenge for participants to program and test different possible solutions to solve it, later transferred to the real scenario with the physical robot and the ramp. The Webots platform was chosen, and a virtual ramp was designed and built on the same scale as the real one to reproduce the challenge as closely as possible to the physical environment. The imported robot reproduces the created prototype without the physics engine component associated with its movement. The virtual world and the robot are represented in Figure 11.

We tested the virtual ramp using robots that already exist in the simulator, like the *Sojourner*, and we noticed that it works perfectly. The robot overtakes the obstacles, although this robot is a bit different in form and motor strength. A prototype of the Vulcano robot working in the simulator will be an added value for the competition.

## 5 Conclusion

In this paper, we present a new robotic challenge, called Vulcano, exhibited for the first time in the second Azorean Robotics Open, Azoresbot 2022. We present the rules as well as the learning goals. We also described the assembly of the ramp where the robot competes and that simulates a volcanic slope, as well as the construction of a new robot prototype that uses whegs capable of climbing the ramp. We analyzed a set of robotic challenges in different application areas, trying to situate our proposal, thus showing that there are some innovative aspects in the Vulcano challenge.

Overall, the implementation of the challenge in Azoresbot 2022 was a success, not only regarding the aspects linked to the robot, developed and improved by the students, but mainly due to the formative issues demonstrated by the teams’ performance, both technological and social and cooperative. All the participants demonstrated this in their comments during the event and in their answers to the questionnaire provided.

However, during the competition, we identified some shortcomings and proposed some changes to the competition in order to provide more complex challenges, for example, forcing teams to add more sensors and to be able to collect more data as they perform the tasks in the challenge scenario.

We hope, in the near future, to test this competition with a larger number of teams. In this regard, we intend to finish the development of the virtual environment to facilitate access to this challenge. This virtual environment will allow teams to test their programs without having access to the platform and may, in the future, support a virtual competition.

## Data Availability

The original contributions presented in the study are included in the article/supplementary material, further inquiries can be directed to the corresponding author.
